# A nomogram based on radiomics signature and deep-learning signature for preoperative prediction of axillary lymph node metastasis in breast cancer

**DOI:** 10.3389/fonc.2022.940655

**Published:** 2022-10-20

**Authors:** Dawei Wang, Yiqi Hu, Chenao Zhan, Qi Zhang, Yiping Wu, Tao Ai

**Affiliations:** ^1^ Department of Plastic Surgery, Tongji Hospital, Tongji Medical College, Huazhong University of Science and Technology, Wuhan, China; ^2^ Department of Radiology, Tongji Hospital, Tongji Medical College, Huazhong University of Science and Technology, Wuhan, China

**Keywords:** breast cancer, axillary lymph node metastasis, radiomics, deep learning, prediction

## Abstract

**Purpose:**

To develop a nomogram based on radiomics signature and deep-learning signature for predicting the axillary lymph node (ALN) metastasis in breast cancer.

**Methods:**

A total of 151 patients were assigned to a training cohort (n = 106) and a test cohort (n = 45) in this study. Radiomics features were extracted from DCE-MRI images, and deep-learning features were extracted by VGG-16 algorithm. Seven machine learning models were built using the selected features to evaluate the predictive value of radiomics or deep-learning features for the ALN metastasis in breast cancer. A nomogram was then constructed based on the multivariate logistic regression model incorporating radiomics signature, deep-learning signature, and clinical risk factors.

**Results:**

Five radiomics features and two deep-learning features were selected for machine learning model construction. In the test cohort, the AUC was above 0.80 for most of the radiomics models except DecisionTree and ExtraTrees. In addition, the K-nearest neighbor (KNN), XGBoost, and LightGBM models using deep-learning features had AUCs above 0.80 in the test cohort. The nomogram, which incorporated the radiomics signature, deep-learning signature, and MRI-reported LN status, showed good calibration and performance with the AUC of 0.90 (0.85-0.96) in the training cohort and 0.90 (0.80-0.99) in the test cohort. The DCA showed that the nomogram could offer more net benefit than radiomics signature or deep-learning signature.

**Conclusions:**

Both radiomics and deep-learning features are diagnostic for predicting ALN metastasis in breast cancer. The nomogram incorporating radiomics and deep-learning signatures can achieve better prediction performance than every signature used alone.

## Introduction

Breast cancer is the most common cancer worldwide that seriously threatens women’s health and survival (1). Axillary lymph node (ALN) status is a valuable prognostic factor and strongly correlated with breast cancer staging, therapy decision-making, distant recurrence, and overall survival rate (2). Clinically, sentinel lymph node biopsy (SLNB) and axillary lymph node dissection (ALND) are routine methods for assessing ALN status. However, these invasive procedures can lead to potential complications such as arm pain, lymphedema, numbness, and seroma (3). Therefore, it is beneficial to develop an accurate and non-invasive approach for assessing ALN status preoperatively to reduce the unnecessary lymph node dissection.

Dynamic contrast-enhanced magnetic resonance imaging (DCE-MRI) has been accepted as a routine imaging modality in evaluating breast cancer because of its ability to reflect the angiogenesis of tumors by injecting contrast agents (4). Previous studies have investigated ALN status with morphological features on MRI, such as node shape and size, cortical thickness, the fatty hilum, disappearance of lymph parenchyma, and enhancement patterns (5–7). However, a high false-negative rate remains a significant problem for the preoperative prediction of ALN metastasis because of the limited power of these traditional clinical and imaging characteristics.

Radiomics analysis has been widely applied in diagnosing, identifying molecular subtypes, and predicting breast cancer chemotherapy response (8–10). Several studies have utilized radiomics and machine learning algorithms to predict ALN metastasis with acceptable results (11–13). Recently, deep learning has progressed in various classification and recognition tasks (14, 15) and has also been proposed for predicting ALN metastasis in breast cancer (16, 17). In addition, deep learning features extracted from pre-processed MR images have been of diagnostic value for ALN metastasis (18, 19). Radiomics features are artificially defined features, while deep learning features are extracted by a convolutional neural network (CNN). A model combining radiomics signature and deep learning signature was reported to be promising to predict LN metastasis in lung cancer (20). However, few studies used both radiomics and deep learning to predict ALN metastasis in breast cancer (16).

Therefore, this study aimed to assess the effectiveness of radiomics features and deep learning features for predicting ALN metastasis, and to develop and validate a nomogram based on radiomics signature, deep learning signature, and clinical factors.

## Materials and methods

### Patients

This retrospective study was approved by the institutional review board of Tongji Hospital (TJ-IRB20220411). We retrospectively reviewed the patients with breast cancer who were treated in our hospital between January 2014 and January 2019. Inclusion criteria were as follows: (a) patients with pathologically confirmed invasive breast cancer; (b) patients with ALN status determined by axillary lymph node dissection (ALND); (c) breast DCE-MRI performed within two weeks before breast surgery. Exclusion criteria were as follows: (a) a history of preoperative therapy including radiotherapy or neoadjuvant chemotherapy; (b) poor image tumor segmentation. Finally, a total of 151 patients were enrolled in this study. The enrolled patients were divided into two cohorts: 106 patients treated between January 2014 and June 2018 were assigned to a training cohort, and 45 patients treated between July 2018 and January 2019 were assigned to a test cohort.

### Clinical and pathological characteristics

Clinical and pathological characteristics were obtained from the electronic medical records of the Hospital Information System (HIS), including patient age, menstrual status, tumor size, histological type, ALN metastasis status, and status of estrogen receptor (ER), progesterone receptor (PR), human epidermal growth factor receptor 2 (HER-2), and Ki-67. ER or PR positive was defined as 10% or more immunostained cells, and HER-2 positive was defined as at least 3+ in Hematoxylin-eosin staining. Ki-67 with a proliferation index higher than 14% was considered positive. ALN with a short diameter greater than 10mm, circular shape, missing fatty hilum, or eccentric cortical thickening were regarded as MRI-reported LN-positive (21).

### MRI image acquisition

Breast MRI images were obtained by a 3.0T scanner (Skyra, Siemens Healthcare, Erlangen, Germany) using a dedicated 16-channel phased-array breast coil in the prone position. T1-weighted DCE-MRI images were analyzed in this study, using a TWIST-VIBE sequence: repetition time (TR) 5.24 ms, echo time (TE) 2.46 ms, matrix size 182 x 320, slice thickness 1.5 mm, FOV 260 x 320 mm2, flip angle 10°, temporal resolution 5.94 sec/phase, and the total scan time 5min57sec. The contrast medium (Omniscan, GE Healthcare, Milwaukee, WI) was injected at the end of the third acquisition phase with a dose of 0.1 mmol/kg body weight, then followed by a 20 ml saline flush at a rate of 2.5 mL/s.

### Workflow

**Figure 1:**
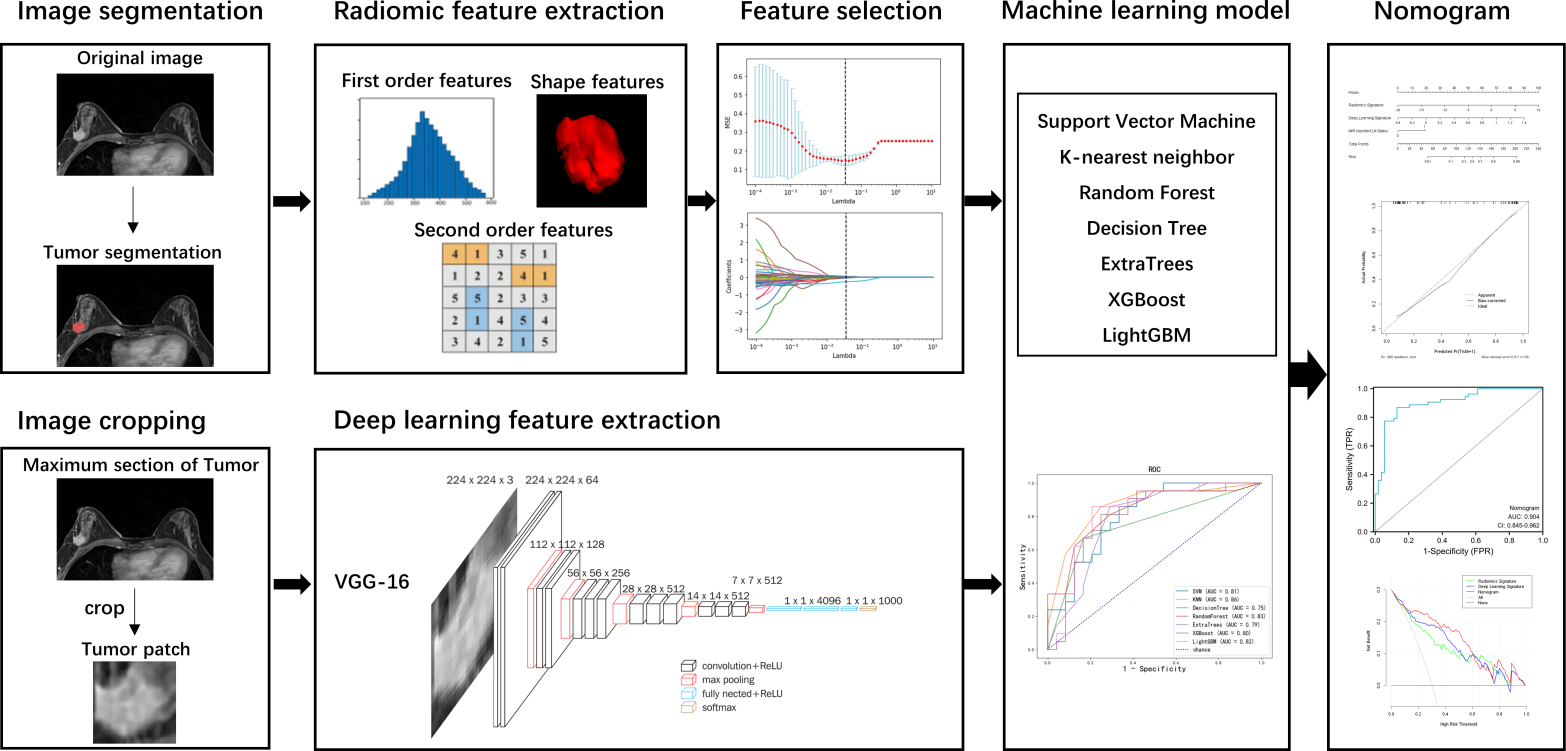
Study workflow.

The workflow of this study is illustrated in **Figure 1**, consisting of tumor segmentation, radiomics and deep-learning features extraction, feature selection, machine learning model development, nomogram construction, and performance assessment.

### Image segmentation

Manual segmentation of the tumor’s three-dimensional region of interest (ROI) was performed on the axial DCE-MRI images using the ITK-SNAP software (http://www.itksnap.org). The ROI was drawn along the tumor’s outline to include the whole lesion without the information about the LN status. The largest tumor lesion was segmented for the patients with multiple lesions in the breast. The ROI was manually segmented by a radiologist with 5 years of experience who was blinded to the lymph node status. The ROI was then confirmed and adjusted by a senior radiologist to ensure the accuracy of the segmentation.

### Radiomic feature extraction

Radiomic features were extracted using open-source extraction software Pyradiomics (http://pyradiomics.readthedocs.io) (22). A total of 120 radiomics features were extracted from each lesion, including 19 first-order features, 16 shape features (3D), 10 shape features (2D), 24 gray level co-occurrence matrix (GLCM) features, 16 gray level size zone matrix (GLSZM) features, 16 gray level run length matrix (GLRLM) features, 5 neighboring gray-tone difference matrix (NGTDM) features, and 14 gray level dependence matrix (GLDM) features.

### Deep-learning feature extraction

VGG-16 was used to extract deep-learning features (23, 24). The maximum cross-sectional area of the tumor ROI was selected and cropped to the two-dimensional rectangular image covering the entire tumor. The tumor patch was re-sized to 224 × 224 and input to the VGG-16 model. For the pre-trained VGG-16 on the ImageNet dataset, transfer learning finetuned the model with the data of our training cohort to adjust the weights of the model. The VGG-16 models were trained up to 100 epochs with 16 mini-batch sizes. In order to make the transfer learning model easier to converge and reduce overfitting, online data augmentation was used to increase the amount of data. The cropped images underwent random horizontal and vertical flipping, rotation, and displacement to achieve data augmentation. After data was input into the trained VGG-16, the features of the last full connection layer of VGG-16 were extracted as deep-learning features.

### Feature selection and machine learning model development

A two-stage feature selection was performed to reduce the irrelevant and acquire the most relevant features. First, t-tests were performed to select the features with p < 0.05. Then, the least absolute shrinkage and selection operator (LASSO) logistic regression algorithm was used to select the most optimal predictive features in the training cohort.

Seven machine learning models were built using the selected features, including Support Vector Machine (SVM), K-nearest neighbor (KNN), RandomForest, DecisionTree, ExtraTrees, XGBoost, and LightGBM. The 5-fold cross-validation method was used to verify the predictive performance of each model in the training cohort. Then, an independent test cohort was further tested to validate the performance of the seven models. The performance of the models was evaluated by the receiver operating characteristic (ROC) curve and the area under the curve (AUC). Accuracy, precision, recall, specificity, and F1-score were also calculated.

### Nomogram construction and performance assessment

Radiomics and deep learning signature were calculated by the linear combination of selected features weighted by LASSO coefficients. Then, a nomogram was constructed based on the multivariate logistic regression model incorporating radiomics signature and deep-learning signature. The performance of the nomogram was assessed with the ROC and AUC values. The calibration of the nomogram was evaluated using a calibration curve and Hosmer-Lemeshow test. Decision curve analysis (DCA) was adopted to estimate the net benefits at different threshold probabilities in the total cohort.

### Statistical analysis

The statistical analyses of this study were performed using Python 3.6 and R 3.5. In order to compare the difference between ALN-Negative and ALN-Positive groups, student’s t-test or Mann-Whitney U test was used for quantitative variables, and the chi-square test or Fisher’s exact test was used for categorical variables. P < 0.05 was considered statistically significant.

## Results

### Clinical and pathological characteristics

The characteristics in the training and test cohorts are listed in **Table 1**. There were no significant differences between the ALN-Negative and ALN-Positive groups regarding age, tumor size, histological type, ER, PR, HER-2, and Ki-67 status (all P > 0.05). Statistical difference was observed in MRI-reported LN status between the ALN-Negative and ALN-Positive groups (P < 0.001).

**Table 1:** Clinical characteristics in training and test cohorts.

Characteristics	Training Cohort (n=106)			Test Cohort (n=45)		
	ALN-Negative (n=54)	ALN-Positive (n=52)	P	ALN-Negative (n=24)	ALN-Positive (n=21)	P
Age (Mean±SD), years	44.50±9.25	43.64±9.65	0.638	45.38±9.00	44.24±9.15	0.677
Tumor size (Mean±SD), cm	3.08±1.81	3.04±1.32	0.905	2.69±1.68	3.60±1.81	0.091
Histological type			0.206			0.083
Invasive ductal carcinoma	49 (90.7%)	51 (98.1%)		23 (95.8%)	16 (76.2%)	
Others	5 (9.3%)	1 (1.9%)		1 (4.2%)	5 (23.8%)	
ER status			0.982			0.339
Negative	24 (44.4%)	23 (44.2%)		7 (29.2%)	9 (42.9%)	
Positive	30 (55.6%)	29 (55.8%)		17 (70.8%)	12 (57.1%)	
PR status			0.712			0.936
Negative	31 (57.4%)	28 (53.8%)		10 (41.7%)	9 (42.9%)	
Positive	23 (42.6%)	24 (46.2%)		14 (58.3%)	12 (57.1%)	
HER-2 status			0.618			0.330
Negative	36 (66.7%)	37 (71.2%)		16 (66.7%)	17 (81.0%)	
Positive	18 (33.3%)	15 (28.8%)		8 (33.3%)	4 (19.0%)	
Ki-67 status			0.545			0.503
Negative	13 (24.1%)	10 (19.2%)		7 (29.2%)	4 (19.0%)	
Positive	41 (75.9%)	42 (80.8%)		17 (70.8%)	17 (81.0%)	
MRI-reported LN status			< 0.001			< 0.001
ALN-Negative	39 (72.2%)	21 (40.4%)		19 (79.2%)	5 (23.8%)	
ALN-Positive	15 (27.8%)	31 (59.6%)		5 (20.8)	16 (76.2%)	

### Prediction performance of radiomics features

**Figure 2:**
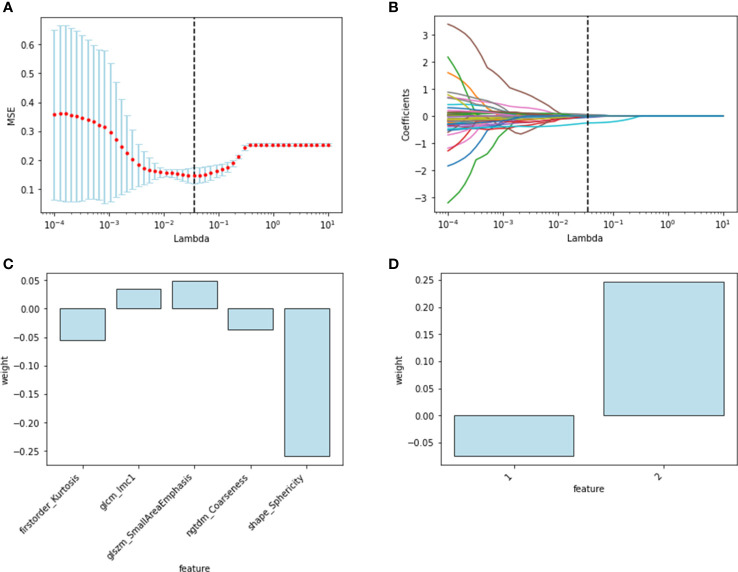
Radiomics and deep-learning feature selection. Radiomics feature selection using LASSO logistic regression: **(A)** selection of the tuning parameter; **(B)** LASSO coefficient profiles of the radiomics features. The final selected features with weights: **(C)** radiomics features; **(D)** deep learning features.

**Figure 3:**
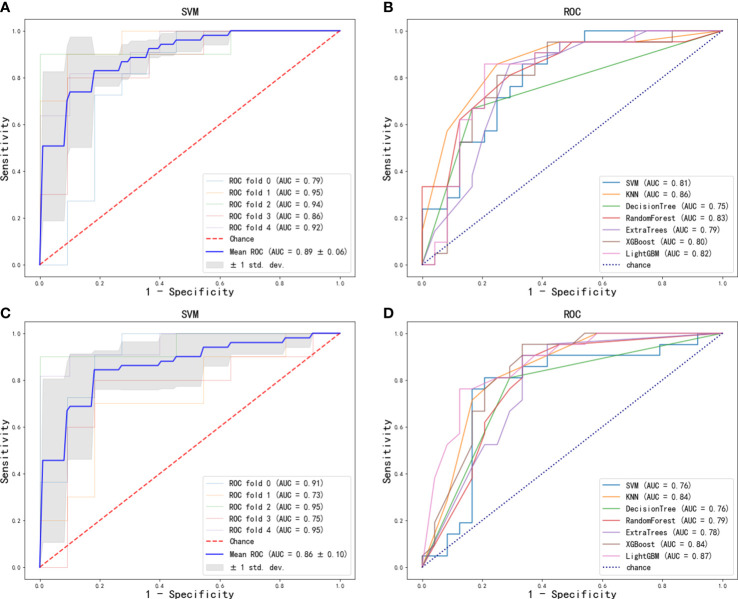
ROC curve of radiomics features for the prediction of ALN metastasis: **(A)** optimal model (SVM) in training cohort; **(B)** seven machine learning models in test cohort. ROC curve of deep-learning features for the prediction of ALN metastasis: **(C)** optimal model (SVM) in training cohort; **(D)** seven machine learning models in test cohort.

One hundred twenty radiomics features were extracted from the DCE-MRI images of the training cohort, which were further reduced to five ALN status-related features using LASSO logistic regression (**Figures 2A** **–C**). We used 7 classifiers to construct machine learning models with radiomics features. The performance of different classifiers is shown in **Table 2** and **Figures 3A** **, B**. The SVM model had the best performance in the training cohort with an AUC of 0.89 (0.84-0.94). In the test cohort, the AUC values of SVM, KNN, DecisionTree, RandomForest, ExtraTrees, XGBoost and LightGBM were 0.81, 0.86, 0.75, 0.83, 0.79, 0.80, and 0.82, respectively.

**Table 2:** The performance of radiomics features for the prediction of ALN metastasis.

Model	AUC	Accuracy	Precision	Recall	F1-Score
Training Cohort					
SVM	0.89 (0.84-0.94)	0.82 (0.75-0.89)	0.84 (0.73-0.95)	0.81 (0.74-0.88)	0.82 (0.76-0.88)
KNN	0.83 (0.77-0.89)	0.71 (0.64-0.78)	0.68 (0.62-0.74)	0.78 (0.65-0.91)	0.72 (0.64-0.80)
DecisionTree	0.71 (0.63-0.79)	0.71 (0.63-0.79)	0.69 (0.60-0.79)	0.75 (0.66-0.84)	0.72 (0.64-0.80)
RandomForest	0.86 (0.80-0.92)	0.78 (0.74-0.82)	0.81 (0.70-0.92)	0.77 (0.71-0.83)	0.78 (0.75-0.82)
ExtraTrees	0.87 (0.82-0.92)	0.77 (0.69-0.85)	0.82 (0.69-0.95)	0.74 (0.63-0.85)	0.76 (0.67-0.85)
XGBoost	0.84 (0.78-0.90)	0.78 (0.69-0.87)	0.77 (0.67-0.87)	0.81 (0.69-0.93)	0.79 (0.70-0.88)
LightGBM	0.84 (0.80-0.88)	0.74 (0.64-0.84)	0.73 (0.63-0.83)	0.76 (0.62-0.90)	0.73 (0.62-0.84)
Test Cohort					
SVM	0.81	0.76	0.69	0.86	0.77
KNN	0.86	0.80	0.75	0.96	0.8
DecisionTree	0.75	0.76	0.78	0.67	0.72
RandomForest	0.83	0.76	0.71	0.81	0.76
ExtraTrees	0.79	0.73	0.71	0.71	0.71
XGBoost	0.8	0.76	0.73	0.76	0.74
LightGBM	0.82	0.82	0.78	0.86	0.82

### Prediction performance of deep-learning features

The features of the last fully connected layer of VGG-16 were weighted by transfer learning and further reduced to two ALN status-related deep-learning features (**Figure 2D**). We also used 7 classifiers to construct machine learning models with deep-learning features. The performance of different classifiers is shown in **Table 3** and **Figures 3C** **, D**. The SVM model had the best performance in the training cohort with an AUC of 0.86 (0.77-0.95). In the test cohort, the AUC values of SVM, KNN, DecisionTree, RandomForest, ExtraTrees, XGBoost and LightGBM were 0.76, 0.84, 0.76, 0.79, 0.78, 0.84, and 0.87, respectively.

**Table 3:** The performance of deep-learning features for the prediction of ALN metastasis.

Model	AUC	Accuracy	Precision	Recall	F1-Score
Training Cohort					
SVM	0.86 (0.77-0.95)	0.80 (0.75-0.85)	0.82 (0.72-0.92)	0.79 (0.73-0.85)	0.80 (0.75-0.85)
KNN	0.84 (0.76-0.92)	0.82 (0.76-0.88)	0.84 (0.75-0.93)	0.79 (0.73-0.85)	0.81 (0.74-0.88)
DecisionTree	0.74 (0.68-0.80)	0.75 (0.68-0.82)	0.77 (0.66-0.88)	0.71 (0.63-0.79)	0.73 (0.66-0.80)
RandomForest	0.79 (0.73-0.85)	0.73 (0.65-0.81)	0.74 (0.65-0.83)	0.69 (0.56-0.82)	0.71 (0.61-0.81)
ExtraTrees	0.76 (0.68-0.84)	0.73 (0.64-0.82)	0.74 (0.64-0.84)	0.67 (0.53-0.81)	0.70 (0.59-0.81)
XGBoost	0.83 (0.78-0.88)	0.74 (0.68-0.80)	0.74 (0.66-0.82)	0.71 (0.60-0.82)	0.72 (0.64-0.80)
LightGBM	0.85 (0.78-0.92)	0.82 (0.77-0.87)	0.86 (0.77-0.95)	0.77 (0.73-0.81)	0.81 (0.76-0.86)
Test Cohort					
SVM	0.76	0.78	0.74	0.81	0.77
KNN	0.84	0.78	0.74	0.81	0.77
DecisionTree	0.76	0.76	0.71	0.81	0.76
RandomForest	0.79	0.73	0.7	0.76	0.73
ExtraTrees	0.78	0.69	0.65	0.71	0.68
XGBoost	0.84	0.78	0.72	0.86	0.79
LightGBM	0.87	0.78	0.74	0.81	0.77

### Nomogram construction and performance assessment

**Figure 4:**
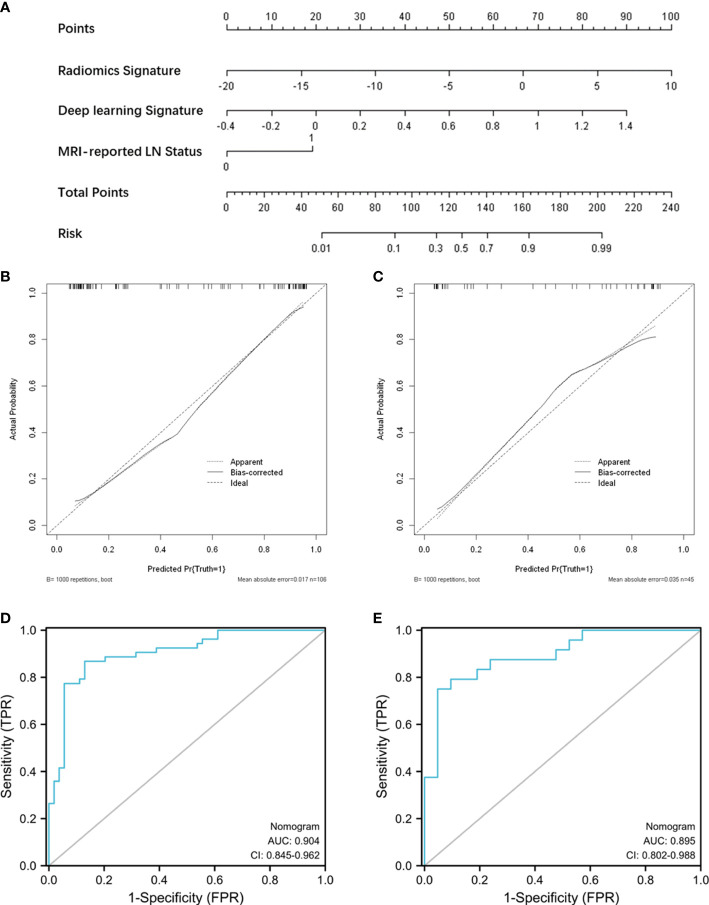
Nomogram construction and performance assessment. **(A)** Nomogram for prediction of ALN metastasis using the radiomics signature, deep-learning signature, and MRI-reported LN status. Calibration curve of the nomogram for the training cohort **(B)** and test cohort **(C)**. ROC curve of the nomogram for the training cohort **(D)** and test cohort **(E)**.

**Figure 5:**
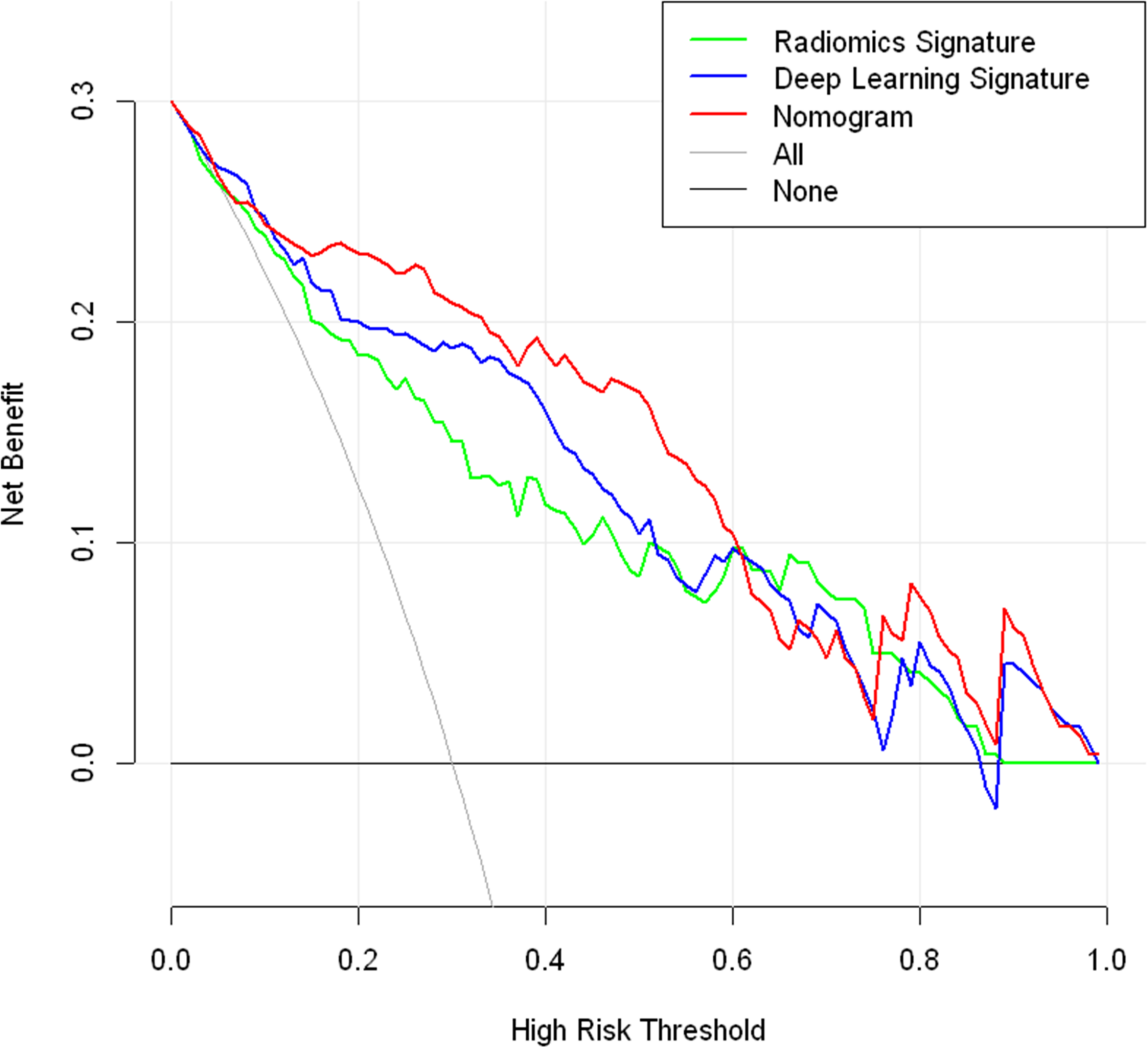
Decision curve analysis of the nomogram.

Radiomic signature and deep learning signature were constructed by the linear combination of selected features respectively (**Figures 2C** **, D**). A nomogram based on radiomics signature, deep-learning signature, and MRI-reported LN status was developed (**Figure 4A**). The calibration curve of the nomogram showed a good agreement between prediction and observation in the training cohort (P = 0.345, **Figure 4B**) and test cohort (P = 0.541, **Figure 4C**). The nomogram displayed an AUC of 0.90 (0.85-0.96) in the training cohort (**Figure 4D**) and 0.90 (0.80-0.99) in the test cohort (**Figure 4E**). The DCA showed that the nomogram predicting ALN metastasis would benefit more than treat-all or treat-none strategy when the threshold probability was greater than 0.10 (**Figure 5**). Besides, the nomogram offered more net benefit than radiomics signature or deep-learning signature at a threshold probability between 0.14 and 0.60.

## Discussion

In this study, we assessed the prediction performance of radiomics features and deep-learning features from DCE-MRI for predicting ALN status in breast cancer. Our results showed that both radiomics and deep-learning features performed well in predicting ALN metastasis. Moreover, we developed a nomogram based on a radiomics signature, deep learning signature, and MRI-reported LN status to predict the ALN status. The results demonstrated that the combination of radiomics and deep-learning signatures improved the predictive performance with an AUC of 0.90 in the test cohort.

Radiomics can convert medical images into extensive quantitative data, which can be used for disease diagnosis, treatment, or prognosis (8, 9). Several studies have used DCE-MRI to predict ALN metastasis with promising results (25–29). Arefan et al. compared the effects of two-dimensional and three-dimensional analysis of radiomics, achieving good prediction performance (25). The prediction performance of the ALN status can be improved by combining the peri-tumoral and intra-tumoral features (26). Similarly, the combination of radiomics with clinical parameters or pharmacokinetic parameters can yield more accurate predictions (27, 28). In addition, studies indicated that different machine learning models could influence prediction performance (25, 29). In this study, the AUC was above 0.80 for most radiomics models except DecisionTree and ExtraTrees, similar to the previously reported results (25, 29). We found that SVM had the best predictive performance in the training cohort, while the KNN had the largest AUC in the test cohort. Despite the good performance of radiomics for predicting ALN metastasis, the shortcoming of radiomics involves the manual drawing of tumor regions, which is relatively time-consuming and subjective.

Deep learning is another feasible technique for predicting ALN metastasis in breast cancer (30–33). Luo et al. extracted deep-learning features from MR images and classified them with SVM, which achieved remarkable prediction performance with an AUC of 0.852 (30). The combined features with multiparametric MRI or multiple views can improve the prediction performance of deep learning (19, 31). Furthermore, deep learning can directly classify ALN status using pre-processed MRI images (32, 33). End-to-end deep learning models generally require a large amount of training data to achieve favorable prediction results. In this study, we extracted deep-learning features from the DCE-MRI images of 151 patients included, and then developed machine learning models to predict ALN metastasis. Our results also demonstrated the value of deep-learning features in diagnosing ALN metastasis. The KNN, XGBoost, and LightGBM models had achieved AUCs above 0.80 in the test cohort. The SVM exhibited an overfitting problem, reflected in an AUC of 0.86 in the training cohort but only 0.76 in the test cohort. However, the DecisionTree and ExtraTrees exhibited underfitting, which may be due to the simplicity of the trained model.

The CNN automatically learns the deep learning features, while the handcrafted radiomics features are artificially defined. In contrast with the low-order image features of radiomics, deep learning extracts high-level features from image patches in a data-driven way. Meanwhile, unlike radiomics requiring complicated manual outlining of tumor regions, deep learning needs to pre-process images into small two-dimensional patches containing the largest cross-section of tumors (18, 20). A fixed-size bounding box covering the entire tumor region was used for deep-learning feature extraction, which could provide both intra-tumoral and peri-tumoral information. Therefore, deep-learning features could complement predictive information to improve the prediction performance of radiomics features.

Considering the influence of clinical factors on ALN metastasis, we developed a nomogram incorporating a radiomics signature, deep-learning signature, and MRI-reported LN status. The nomogram displayed good calibration and excellent performance to evaluate LN status with an AUC of 0.90 (0.85-0.96) in the training cohort and 0.90 (0.80-0.99) in the test cohort. The DCA also showed that the nomogram yielded more net benefits than a single signature to predict ALN metastasis in clinical use preoperatively. The performance of the nomogram in this study was not inferior to the reported models for predicting ALN metastasis. Han et el. developed a radiomic nomogram based on a radiomic signature and clinical features, resulting in the AUCs of 0.84 and 0.87 in training and validation cohorts (27). Similarly, Song et al. established a nomogram incorporating the histological grade, multifocality, radiomics signature, and MR-reported ALN status, showing good performance in the validation set with an AUC of 0.874 (34). In our results, adding deep-learning signature to the nomogram model had the potential to further improve predictive performance.

There are some limitations to this study. First, this study focused on the primary tumor features rather than those of the lymph nodes. Positive ALNs are not always visualized on breast MRI, and it isn’t easy to match the biopsied ALNs to those imaged on MRI for multiple lymph nodes. Second, the tumor segmentation was manually performed, which could be impacted by the radiologist’s experience. Automated segmentation is required for the objective assessment of ALN metastasis. Third, this was a single-center retrospective study with relatively small samples. Small training samples tend to lead to overfitting of the model. Studies with larger sample sizes from different centers are needed to validate our findings in further investigation.

In conclusion, this study confirmed the diagnostic value of radiomics and deep-learning features based on DCE-MRI for predicting ALN metastasis. We also developed a nomogram based on a radiomics signature, deep-learning signature, and MRI-reported LN status, achieving a better prediction performance in the test cohort. This study could provide a highly effective, non-invasive method for the preoperative prediction of ALN metastasis, assisting the personalized treatment strategies for patients with breast cancer.

## Data availability statement

The raw data supporting the conclusions of this article will be made available by the authors, without undue reservation.

## Ethics statement

This retrospective study was approved by the institutional review board of Tongji Hospital (TJ-IRB20220411). The ethics committee waived the requirement of written informed consent for participation.

## Author contributions

DW, TA, and YW participated in the conception and design of the study. YH and CZ collected the clinical and imaging data. DW and QZ performed the statistical analyses. DW, QZ, and TA coordinated, drafted, revised and finalized the manuscript. All authors contributed to the article and approved the submitted version.

## Conflict of interest

The authors declare that the research was conducted in the absence of any commercial or financial relationships that could be construed as a potential conflict of interest.

## Publisher’s note

All claims expressed in this article are solely those of the authors and do not necessarily represent those of their affiliated organizations, or those of the publisher, the editors and the reviewers. Any product that may be evaluated in this article, or claim that may be made by its manufacturer, is not guaranteed or endorsed by the publisher.

## Abbreviations

ALN, Axillary lymph node; ALND, Axillary lymph node dissection; AUC, Area under the curve; CNN, Convolutional neural network; DCE-MRI, Dynamic contrast-enhanced magnetic resonance imaging; ER, Estrogen receptor; HER-2, Human epidermal growth factor receptor 2; KNN, K-nearest neighbor; LASSO, Least absolute shrinkage and selection operator; PR, Progesterone receptor; ROC, Receiver operating characteristic; ROI, Region of interest; SLNB, Sentinel lymph node biopsy; SVM, Support Vector Machine; TE, Echo time; TR, Repetition time.

## References

[1] Fan L Strasser-Weippl K Li J-J St Louis J Finkelstein DM Yu K-D. Breast cancer in China. Lancet Oncol (2014) 15:e279–89. doi: 10.1016/S1470-2045(13)70567-9 24872111

[2] Pilewskie M Morrow M. Axillary nodal management following neoadjuvant chemotherapy: A review. JAMA Oncol (2017) 3:549–55. doi: 10.1001/jamaoncol.2016.4163 PMC5580251 27918753

[3] Sakorafas GH Peros G Cataliotti L. Sequelae following axillary lymph node dissection for breast cancer. Expert Rev Anticancer Ther (2006) 6:1629–38. doi: 10.1586/14737140.6.11.1629 17134366

[4] Turnbull LW. Dynamic contrast-enhanced MRI in the diagnosis and management of breast cancer. NMR BioMed (2009) 22:28–39. doi: 10.1002/nbm.1273 18654999

[5] Schipper R-J Paiman M-L Beets-Tan RGH Nelemans PJ de Vries B Heuts EM. Diagnostic performance of dedicated axillary T2- and diffusion-weighted MR imaging for nodal staging in breast cancer. Radiology (2015) 275:345–55. doi: 10.1148/radiol.14141167 25513854

[6] Choi EJ Youk JH Choi H Song JS. Dynamic contrast-enhanced and diffusion-weighted MRI of invasive breast cancer for the prediction of sentinel lymph node status. J Magn Reson Imaging (2020) 51:615–26. doi: 10.1002/jmri.26865 31313393

[7] Scaranelo AM Eiada R Jacks LM Kulkarni SR Crystal P. Accuracy of unenhanced MR imaging in the detection of axillary lymph node metastasis: study of reproducibility and reliability. Radiology (2012) 262:425–34. doi: 10.1148/radiol.11110639 22143924

[8] Conti A Duggento A Indovina I Guerrisi M Toschi N. Radiomics in breast cancer classification and prediction. Semin Cancer Biol (2021) 72:238–50. doi: 10.1016/j.semcancer.2020.04.002 32371013

[9] Liu Z Li Z Qu J Zhang R Zhou X Li L. Radiomics of multiparametric MRI for pretreatment prediction of pathologic complete response to neoadjuvant chemotherapy in breast cancer: A multicenter study. Clin Cancer Res (2019) 25:3538–47. doi: 10.1158/1078-0432.CCR-18-3190 30842125

[10] Osman AFI. A multi-parametric MRI-based radiomics signature and a practical ML model for stratifying glioblastoma patients based on survival toward precision oncology. Front Comput Neurosci (2019) 13:58. doi: 10.3389/fncom.2019.00058 31507398 PMC6718726

[11] Yu Y He Z Ouyang J Tan Y Chen Y Gu Y. Magnetic resonance imaging radiomics predicts preoperative axillary lymph node metastasis to support surgical decisions and is associated with tumor microenvironment in invasive breast cancer: A machine learning, multicenter study. EBioMedicine (2021) 69:103460. doi: 10.1016/j.ebiom.2021.103460 34233259 PMC8261009

[12] Zhang X Yang Z Cui W Zheng C Li H Li Y. Preoperative prediction of axillary sentinel lymph node burden with multiparametric MRI-based radiomics nomogram in early-stage breast cancer. Eur Radiol (2021) 31:5924–39. doi: 10.1007/s00330-020-07674-z 33569620

[13] Samiei S Granzier RWY Ibrahim A Primakov S Lobbes MBI Beets-Tan RGH. Dedicated axillary mri-based radiomics analysis for the prediction of axillary lymph node metastasis in breast cancer. Cancers (Basel) (2021) 13:1–15. doi: 10.3390/cancers13040757 PMC7917661 33673071

[14] Yala A Lehman C Schuster T Portnoi T Barzilay R. A deep learning mammography-based model for improved breast cancer risk prediction. Radiology (2019) 292:60–6. doi: 10.1148/radiol.2019182716 31063083

[15] Mambou SJ Maresova P Krejcar O Selamat A Kuca K. Breast cancer detection using infrared thermal imaging and a deep learning model. Sensors (Basel) (2018) 18:2799. doi: 10.3390/s18092799 PMC6164870 30149621

[16] Zheng X Yao Z Huang Y Yu Y Wang Y Liu Y. Deep learning radiomics can predict axillary lymph node status in early-stage breast cancer. Nat Commun (2020) 11:1–9. doi: 10.1038/s41467-020-15027-z 32144248 PMC7060275

[17] Zhou L-Q Wu X-L Huang S-Y Wu G-G Ye H-R Wei Q. Lymph node metastasis prediction from primary breast cancer US images using deep learning. Radiology (2020) 294:19–28. doi: 10.1148/radiol.2019190372 31746687

[18] Yang X Wu L Ye W Zhao K Wang Y Liu W. Deep learning signature based on staging CT for preoperative prediction of sentinel lymph node metastasis in breast cancer. Acad Radiol (2020) 27:1226–33. doi: 10.1016/j.acra.2019.11.007 31818648

[19] Wang Z Sun H Li J Chen J Meng F Li H. Preoperative prediction of axillary lymph node metastasis in breast cancer using CNN based on multiparametric MRI. J Magn Reson Imaging (2022) 56:700–709. doi: 10.1002/jmri.28082 35108415

[20] Ran J Cao R Cai J Yu T Zhao D Wang Z. Development and validation of a nomogram for preoperative prediction of lymph node metastasis in lung adenocarcinoma based on radiomics signature and deep learning signature. Front Oncol (2021) 11:585942. doi: 10.3389/fonc.2021.585942 33968715 PMC8101496

[21] Kim EJ Kim SH Kang BJ Choi BG Song BJ Choi JJ. Diagnostic value of breast MRI for predicting metastatic axillary lymph nodes in breast cancer patients: diffusion-weighted MRI and conventional MRI. Magn Reson Imaging (2014) 32:1230–6. doi: 10.1016/j.mri.2014.07.001 25072504

[22] van Griethuysen JJM Fedorov A Parmar C Hosny A Aucoin N Narayan V. Computational radiomics system to decode the radiographic phenotype. Cancer Res (2017) 77:e104–7. doi: 10.1158/0008-5472.CAN-17-0339 PMC5672828 29092951

[23] Hong D Zheng Y-Y Xin Y Sun L Yang H Lin M-Y. Genetic syndromes screening by facial recognition technology: VGG-16 screening model construction and evaluation. Orphanet J Rare Dis (2021) 16:344. doi: 10.1186/s13023-021-01979-y 34344442 PMC8336249

[24] Yang H Ni J Gao J Han Z Luan T. A novel method for peanut variety identification and classification by improved VGG16. Sci Rep (2021) 11:15756. doi: 10.1038/s41598-021-95240-y 34344983 PMC8333428

[25] Arefan D Chai R Sun M Zuley ML Wu S. Machine learning prediction of axillary lymph node metastasis in breast cancer: 2D versus 3D radiomic features. Med Phys (2020) 47:6334–42. doi: 10.1002/mp.14538 PMC8581816 33058224

[26] Zhan C Hu Y Wang X Liu H Xia L Ai T. Prediction of axillary lymph node metastasis in breast cancer using intra-peritumoral textural transition analysis based on dynamic contrast-enhanced magnetic resonance imaging. Acad Radiol (2022) 29:S107–15. doi: 10.1016/j.acra.2021.02.008 33712393

[27] Han L Zhu Y Liu Z Yu T He C Jiang W. Radiomic nomogram for prediction of axillary lymph node metastasis in breast cancer. Eur Radiol (2019) 29:3820–9. doi: 10.1007/s00330-018-5981-2 30701328

[28] Liu M Mao N Ma H Dong J Zhang K Che K. Pharmacokinetic parameters and radiomics model based on dynamic contrast enhanced MRI for the preoperative prediction of sentinel lymph node metastasis in breast cancer. Cancer Imaging (2020) 20:1–8. doi: 10.1186/s40644-020-00342-x PMC7493182 32933585

[29] Zhu Y Yang L Shen H. Value of the application of CE-MRI radiomics and machine learning in preoperative prediction of sentinel lymph node metastasis in breast cancer. Front Oncol (2021) 11:757111. doi: 10.3389/fonc.2021.757111 34868967 PMC8640128

[30] Luo J Ning Z Zhang S Feng Q Zhang Y. Bag of deep features for preoperative prediction of sentinel lymph node metastasis in breast cancer. Phys Med Biol (2018) 63:245014. doi: 10.1088/1361-6560/aaf241 30523819

[31] Ren T Lin S Huang P Duong TQ. Convolutional neural network of multiparametric MRI accurately detects axillary lymph node metastasis in breast cancer patients with pre neoadjuvant chemotherapy. Clin Breast Cancer (2022) 22:170–7. doi: 10.1016/j.clbc.2021.07.002 34384696

[32] Ha R Chang P Karcich J Mutasa S Fardanesh R Wynn RT. Axillary lymph node evaluation utilizing convolutional neural networks using MRI dataset. J Digit Imaging (2018) 31:851–6. doi: 10.1007/s10278-018-0086-7 PMC6261196 29696472

[33] Ren T Cattell R Duanmu H Huang P Li H Vanguri R. Convolutional neural network detection of axillary lymph node metastasis using standard clinical breast MRI. Clin Breast Cancer (2020) 20:e301–8. doi: 10.1016/j.clbc.2019.11.009 32139272

[34] Song D Yang F Zhang Y Guo Y Qu Y Zhang X. Dynamic contrast-enhanced MRI radiomics nomogram for predicting axillary lymph node metastasis in breast cancer. Cancer Imaging (2022) 22:17. doi: 10.1186/s40644-022-00450-w 35379339 PMC8981871

